# Impedimetric Sensing: An Emerging Tool for Combating the COVID-19 Pandemic

**DOI:** 10.3390/bios13020204

**Published:** 2023-01-30

**Authors:** Victor Ong, Ali Soleimani, Farbod Amirghasemi, Sina Khazaee Nejad, Mona Abdelmonem, Meisam Razaviyayn, Parisa Hosseinzadeh, Lucio Comai, Maral P. S. Mousavi

**Affiliations:** 1 Alfred E. Mann Department of Biomedical Engineering, Viterbi School of Engineering, University of Southern California, Los Angeles, CA 90089, USA; 2 Daniel J. Epstein Department of Industrial and Systems Engineering, Viterbi School of Engineering, University of Southern California, Los Angeles, CA 90089, USA; 3 Ming Hsieh Department of Electrical and Computer Engineering, Viterbi School of Engineering, University of Southern California, Los Angeles, CA 90089, USA; 4 Department of Computer Science, Viterbi School of Engineering, University of Southern California, Los Angeles, CA 90089, USA; 5 Knight Campus Center Department of Bioengineering, University of Oregon, Eugene, OR 97403, USA; 6 Department of Molecular Microbiology and Immunology, Keck School of Medicine, University of Southern California, Los Angeles, CA 90033, USA

**Keywords:** COVID-19, point of care, electrochemical detection, impedance spectroscopy, paper-based devices, sensors, SARS-CoV-2

## Abstract

The COVID-19 pandemic revealed a pressing need for the development of sensitive and low-cost point-of-care sensors for disease diagnosis. The current standard of care for COVID-19 is quantitative reverse transcriptase polymerase chain reaction (qRT-PCR). This method is sensitive, but takes time, effort, and requires specialized equipment and reagents to be performed correctly. This make it unsuitable for widespread, rapid testing and causes poor individual and policy decision-making. Rapid antigen tests (RATs) are a widely used alternative that provide results quickly but have low sensitivity and are prone to false negatives, particularly in cases with lower viral burden. Electrochemical sensors have shown much promise in filling this technology gap, and impedance spectroscopy specifically has exciting potential in rapid screening of COVID-19. Due to the data-rich nature of impedance measurements performed at different frequencies, this method lends itself to machine-leaning (ML) algorithms for further data processing. This review summarizes the current state of impedance spectroscopy-based point-of-care sensors for the detection of the SARS-CoV-2 virus. This article also suggests future directions to address the technology’s current limitations to move forward in this current pandemic and prepare for future outbreaks.

## 1. Introduction

Early detection of SARS-CoV-2 (the coronavirus responsible for the COVID-19 pandemic), and isolation of infected individual is critical for controlling disease spread. Frequent testing through accessible platforms is an important form of secondary prevention. Paltiel et al. calculated that a test with only 70% sensitivity and 98% specificity would be sufficient to allow universities to safely reopen if widely used at least once per week [1,2,3,4]. This is the reality in most of the developed world in late 2022, where widespread testing is common and accessible and the regular monitoring of health data has been normalized through low-cost test strips and wearable devices. The current gold-standard detection technique for COVID-19 is the qRT-PCR. However, it is complex, costly, reagent-heavy, requires advanced infrastructure and skilled labor, and can take hours to days to retrieve results [1]. These factors make frequent qRT-PCR testing infeasible in low-resource environments, where inadequate infrastructure and socioeconomic disparities are common. The days-long delay between testing and result delivery also means infected individuals can unwittingly spread the disease to others before being notified of a positive test result. The current alternative to qRT-PCR is the RAT. The RAT requires minimal training and can be quickly deployed outside specialized laboratories. However, it suffers from low sensitivity (percent of true infections detected) [1,5]. Additionally, colorimetric tests such as these are only reliable for positive/negative decisions and not for quantitative data, hampering their usefulness for policy decisions. Therefore, much effort has been put into developing rapid, accurate, quantitative, and scalable tests for worldwide pandemic management.

As seen with the conventional RATs, colorimetry is a popular technique for point-of-care disease detection and has demonstrated usefulness in the COVID-19 pandemic [6,7]. However, it requires the use of complex reagents and sample mixing and is non-quantitative without a colorimeter or scanning device. Additionally, the observation of a qualitative color change is subjective and can change dramatically based on lighting, the environment, and the observer.

**Figure 1:**
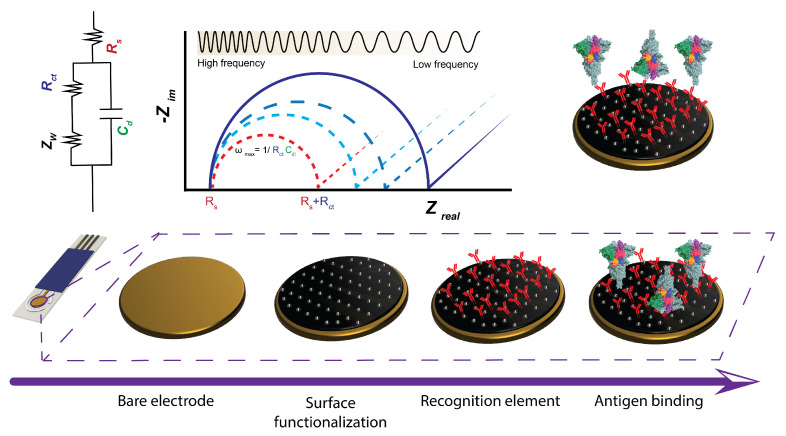
The general concept of electrode fabrication and its effect on a Nyquist plot. Bare electrodes are functionalized, a recognition/capture element is added, and the sensor is exposed to the target. In general, more mass bound to the surface of the electrode increases the semicircular diameter of the Nyquist plot, which is correlated to an increase in $R_{ct}$ in the Randles circuit. However, certain surface modifications can decrease the $R_{ct}$ transfer due to electrostatic interaction at the surface.

Electrochemical detection methods have shown promise for molecular COVID-19 detection due to their rapid action and quantitative results [8,9,10,11,12,13,14,15,16]. Most electrochemical methods are typically label-free, quantitative, objective, and do not require any chemical workup/washing steps [17,18,19,20,21,22]. Among the electrochemical detection methods, electrochemical impedance spectroscopy (EIS) stands out because of its versatility and its data-rich nature. In EIS, electrode impedance is measured at different frequencies in a sample to generate a Nyquist plot, whereas voltammetric and amperometric techniques typically generate a single data point per sample (peak current value). Through immobilization of appropriate capture probes at the electrode surface, EIS can be used to detect a variety of biomarkers with no need for complicated wash steps. This feature makes EIS ideal for point-of-care detection of larger proteins and viruses, such as the SARS-CoV-2 virus (Figure 1).

Previous reviews of COVID-19 diagnostic methods were extremely broad and covered the gamut of different detection methods, analytes, and materials [14,16,23,24,25,26,27,28,29]. Naikoo et al. reviewed nanomaterials for COVID-19 detection, Entesari et al. reviewed detection of coronaviruses in general (including standard-of-care RT-PCR tests and ELISA), Benda et al. reviewed all commercialized technologies for COVID-19 diagnosis (none of which are EIS-based), and Singh et al. provided an overview of every clinical and at-home method for COVID-19 detection, but did not explore EIS biosensors and or perform a detailed discussion of the reviewed papers [23,24,27,28]. Yasri and Wiwanitkit’s review focuses on fabrication techniques and does not go into detail about outcomes, and Kotru et al. reviewed electrochemical sensing as a whole for diagnosing COVID-19, and mentions a plethora of biomarkers such as C-reactive protein, glutamate, and immunoglobulins [25,29]. They also briefly mention direct detection of COVID-19 viral proteins with electrochemical methods, but only touch on two such papers [25]. Similarly, Taha et al.’s general review of biosensors only includes two electrochemical sensors, only one of which pertains to COVID-19 [16]. Saatçi and Natarajan published a review on the use of colloidal particles and interface-based detection methods for diagnosing COVID-19, but only include two papers that use electrochemical sensing to interrogate the sample [26]. Pinheiro et al.’s review only considered a single electrochemical biosensor [14].

These previous reviewes, while useful, do not go into great detail about EIS devices for COVID-19. This review is focused on impedimetric sensors for COVID-19 only, and discusses technical details and specifics of devices, and important future steps in impedimetric sensors for disease diagnosis and management. Here, we aim to survey the current state of EIS sensors for direct COVID-19 detection (detecting either antibodies or portions of the virus itself), compare recent work, and highlight EIS as an agile detection platform for epidemic and pandemic prevention and preparedness. We will also briefly discuss future opportunities to improve the capabilities of impedimetric biosensors through the integration of new classes of capture probes, new materials, and ML tools.

There are numerous variations of EIS-based biosensors for COVID-19. The two main classes of COVID-19 EIS sensors are: (i) those that detect the virus itself (antigen/viral test), and (ii) those that detect antibodies against SARS-CoV-2 (antibody test). Several different capture probes are used, including aptamers, virus-imprinted polymers, proteins, and antibodies [30,31,32,33]. This review will discuss (i) the theory enabling impedimetric sensing, (ii) the anatomy of the SARS-CoV-2 virus, (iii) the design and fabrication of sensors, (iv) detector devices and parameters, (v) analytical performance, (vi) practical consideration for frequent and global COVID-19 detection, and (vii) future perspectives on characterization standards, and novel materials and methods.

## 2. Theoretical Considerations in Impedimetric Sensing

In an EIS biosensor, the surface of a working electrode (WE) in solution forms a double layer of ions that behaves like a capacitor. Changes at the electrode surface (e.g., the selective surface binding of a particular analyte such as the SARS-CoV-2 spike protein) cause measurable differences in the impedance spectra (Figure 1). The electrochemical behavior of the electrode/solution interface is modeled using the Randles equivalent circuit (Figure 1). The Faradaic current (resulting from electron transfer from electrode to a redox active molecule in solution) and non-Faradaic current (capacitive charging of electrode surface) are represented by $R_{ct}$ (charge transfer resistance) and $C_{dl}$ (electrode capacitance). Changes in the electrode impedance are reflected in changes in $R_{ct}$ and $C_{dl}$; a calibration curve can be established to correlate $R_{ct}$, $C_{dl}$, and Δ$R_{ct}$ to biomarker concentration. To understand impedimetric biosensors, it is important to first understand the concept of impedance.

The concept of impedance, introduced by Olivier Heaviside in the 1880s, is the frequency domain ratio of voltage to current [31]. In EIS, a sinusoidal potential is applied to the electrochemical cell and the response, a sinusoidal current, is measured. However, this only holds true for a linear system; to ensure that the electrochemical system is linear, small amplitudes are used [32]. The input voltage has the form of

(1) $$E\left(t\right)=E_{0}sin\left(\omega t\right)$$

where E(t), $E_{0}$, and ω are potential at time *t*, input signal amplitude, and radial frequency, respectively. Since the system is linear, the output signal (measured as current) is a shifted sinusoidal signal with the same radial frequency:

(2) $$I\left(t\right)=I_{0}sin\left(\omega t+\phi \right)$$

where I(t) is the output signal (current), $I_{0}$ is the amplitude of the output signal, ω is the radial frequency, ϕ is the signal phase, and *t* is time.

The impedance of this system is:

(3) $$Z=\frac{E(t)}{I(t)}=\frac{E_{0}sin\left(\omega t\right)}{I_{0}sin\left(\omega t+\phi \right)}=Z_{0}\frac{sin(\omega t)}{sin(\omega t+\phi )}$$

Using Euler’s formula, we can express the impedance as a complex number with amplitude $Z_{0}$ and phase ϕ [33]:

(4) $$Z=Z_{0}e^{j\omega }=Z_{0}\left(cos\phi +jsin\phi \right)$$

Impedance data is often gathered by a frequency sweep and presented in two charts: (1) the Nyquist, and (2) the Bode plots. In the Nyquist plot, the imaginary part of the impedance is plotted against the real part across the frequency range (Figure 1). Each data point is an expression of a single frequency, with higher frequencies on the left and lower frequencies on the right. In the Bode plot, two separate logarithmic charts plot amplitude and phase against frequency.

In order to interpret the Nyquist and Bode plots, an understanding of the elements of a model for EIS system is necessary. An electrochemical cell is modeled with a resistor, constant phase element (CPE), and Warburg element (to represent the effect of mass transport on current).

The resistor is a frequency-independent element; thus, in the Nyquist plot, the amount of resistance does not change with frequency.

(5) $$Z_{R}=R$$

A capacitor is an element in an electrical circuit which stores electric energy. The voltage–current equation of a capacitor is:

(6) $$I\left(t\right)=C\frac{dV}{dt}$$

where *C* is the capacitance and *V* is the applied voltage. If the applied signal to the capacitor is:

(7) $$V\left(t\right)=V_{0}e^{j\omega t}$$

Based on Equation (6), the capacitor current will be:

(8) $$I\left(t\right)=j\omega V_{0}C_{dl}e^{j\omega t}$$

Thus, the impedance of a capacitor (potential over current) is:

(9) $$Z_{C}=\frac{V(t)}{I(t)}=\frac{1}{j\omega C}$$

In electrochemical systems, capacitors are not ideal; therefore, a CPE is used for modeling instead of a capacitor. The impedance of the CPE is:

(10) $$Z_{C}PE=\frac{1}{Q_{0}{\left(j\omega \right)}^{n}}$$

where $Q_{0}$ is the admittance value at ω=1 rad/s, and *n* is a numerical value between 0 and 1. Therefore, unlike the phase of a capacitor which is set at −90${}^{{}^{\circ}}$, the CPE phase is −90${}^{{}^{\circ}}\times n$ [34].

The last element for the modeling is the Warburg element. This is a virtual electronic component which is used for modeling an electrochemical system. In addition to the electrode surface, disruptions or limitations to the mass transport of the redox marker can limit current flow and increase electrode impedance. This resistance is represented differently at different frequencies based on the duration of the pulse, and the time that the diffusion layer grows. Warburg impedance is typically observed at lower frequencies where there is a longer time for growth in the diffusion layer, where mass transport can limit the current flow. The impedance of the Warburg element is:

(11) $$Z_{W}=\frac{\sigma }{\sqrt{\omega }}-j\frac{\sigma }{\sqrt{\omega }}$$

where σ is the Warburg coefficient and is:

(12) $$\sigma =\frac{RT}{n^{2}AF^{2}\sqrt{2}}\left(\frac{1}{C_{O}\sqrt{D_{O}}}+\frac{1}{C_{R}\sqrt{D_{R}}}\right)$$

where *R* and *F* are the gas and Faraday constants, respectivel;, *n* is the number of transferred electrons; *A* is the surface area of the electrode; and *D* and *C* are the diffusion coefficient and bulk concentration of oxidant and reductant. In the Nyquist and Bode plots, the Warburg effect is expressed as a 45${}^{{}^{\circ}}$ line and 45${}^{{}^{\circ}}$ phase shift, respectively.

These electrical components are used to model different phenomena that occur in an EIS measurement. A Randles circuit (Figure 1) is used to model the behavior of an electrochemical system [35]. The circuit is comprised of two resistances, $R_{s}$ and $R_{ct}$, which model the resistance of the solution and resistance to charge transfer at the surface of the electrode; a CPE, which models the behavior of the double layer capacitor ($C_{dl}$); and a Warburg element, which models the diffusion of a redox marker from the bulk of the solution to the surface of the electrode. The elements in this circuit model can change based on a particular EIS system’s properties, including the surface nonuniformities or molecular adsorption or capture; this “change” in the equivalent is used to measure the presence and concentration of analyte.

An EIS system is either kinetically or diffusion-controlled, depending on the frequency. In high frequencies (left side of a Nyquist plot), the system is kinetically controlled. Here, ions in bulk solution do not have enough time to diffuse to the surface of the electrode; thus, the Warburg element does not have any effect [36]. Moreover, at high frequencies, the impedance of the CPE approaches zero, short circuiting this parallel branch of the circuit. Thus, the Nyquist plot shows only the frequency-independent resistance of the solution. As the frequency of the EIS decreases (we move to the right of the Nyquist plot), the effects of the $C_{dl}$ and $R_{ct}$ elements appear. Charge transfer resistance is the diameter of the semi-circle that appears in the Nyquist plot. At low frequencies, the system becomes diffusion-controlled, where the mass transport of redox species dominates the signal. In this region, the effect of the Warburg element is seen in the Nyquist plot as a line with a slope of 45${}^{{}^{\circ}}$. Impedance analysis is commonly used for characterization of electrode surfaces and validation of the immobilization of biomolecules on the electrode surface. The electrode impedance or individual values of $R_{ct}$ and $C_{dl}$ can be utilized as measures of electrode impedance, and, subsequently, on concentration of antigens/biomarkers in a sample solution. Biomarker-specific capture probes (e.g., an antibody) are immobilized on the electrode surface area, resulting in the formation of a complex if an antigen is present within the sample solution, altering electrode impedance. A calibration curve can be established to correlate impedance parameters ($R_{ct}$, $C_{dl}$, and Δ$R_{ct}$) to the biomarker concentration.

## 3. SARS-CoV-2 Anatomy

**Figure 2:**
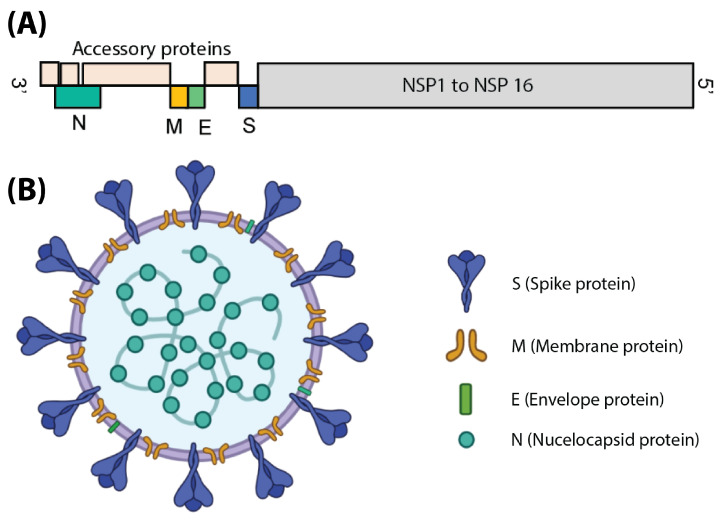
(**A**) The arrangement of the SARS-CoV-2 genome and position of proteins in it. (**B**) The organization of the major structural proteins in the virus particle.

SARS-CoV-2 is a coronavirus from the beta coronavirus 2B lineage [37]. Coronaviruses are a family of RNA viruses that cause disease in mammals and birds. The human diseases that are primarily associated with respiratory tract infections range from the common cold to more severe diseases such as MERS, SARS, and COVID-19. All members of this family have characteristic projections from their surface called spikes, which give them a unique shape under the electron microscope resembling a stellar corona; thus, the name (Figure 2B). Like other coronaviruses, SARS-CoV-2 has an envelope which protects its genome, a positive-sense single-stranded RNA (+ssRNA) with a size of around 29.9 kb [38].

SARS-CoV-2 has four structural proteins (Figure 2B): S (spike protein), E (envelope protein), M (membrane protein), and N (nucleocapsid protein). It also contains 16 non-structural proteins named NSP1 through 16. The non-structural proteins perform a variety of functions essential for viral replication, infection, and life cycle. Some of the most well-known NSPs are NSP12 (RNA-dependent RNA polymerase) and NSP5 (main protease). The N protein is responsible for RNA binding and genome packaging, and, thus, is essential for viral replication. It contains an RNA binding domain as well as a dimerization domain; however, a large part of the protein is predicted to be intrinsically disordered [39].

The membrane is composed of M, E and S proteins in addition to lipids (Figure 2B). The ratio of the E:S:M proteins in coronaviruses is around 1:20:300 [40]. The M proteins are the major structural proteins in SARS-CoV-2 and the most abundant of the three. The SARS-CoV-2 M protein is a 25-30 kDa O-glycosylated protein with three major domains: an N-terminal ectodomain, a transmembrane domain which passes the membrane three times, and a C-terminal endodomain [41]. The M protein is essential in all stages of the viral life cycle, from assembly to budding to infection.

The E proteins are the least abundant proteins on the surface of the virus, with nearly 20 copies present, and have different sequences across species [41]. This small protein of 8.4–12 kDa forms a pentameric ion channel in the membrane through their single-pass alpha helix [41]. The major role of the E protein is in assembly, trafficking, and morphogenesis [42].

**Figure 3:**
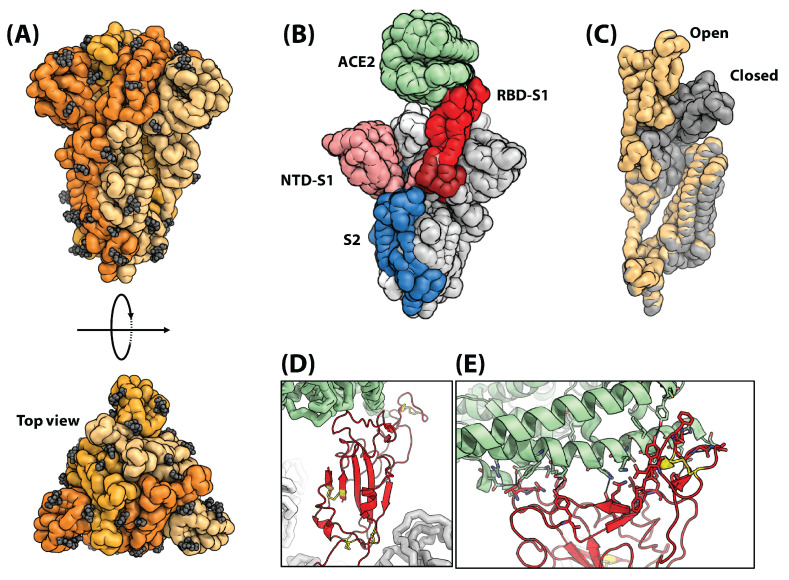
(**A**) Structure of SARS-CoV-2 S protein (PDB ID: 7FCE [45]). The glycosylations are shown as black spheres on the surface of the protein. There are > 20 glycosylation sites in the S protein [46]. (**B**) RBD domain is in charge of binding to ACE2 (PDB ID: 7A97 [47]). (**C**) The S protein can adopt a closed conformation (PDB ID: 7FCE) or an open conformation (PDB ID: 7A97). The open conversation is the one that can engage with ACE2. (**D**,**E**) show the binding interaction of the RBD domain with ACE2. The yellow color indicates disulfide bonds. The dashed lines in E are hydrogen bonds.

The S protein is a glycosylated homotrimer which is in charge of binding to host receptors and causing infection (Figure 3A) [41]. Each monomer is around 150–200 kDa with around 1273 amino acids [41]. In SARS-CoV-2, the spike protein is known to bind to the receptor angiotensin-converting enzyme II (ACE2) [43]. The S protein is subdivided into two components (Figure 3B): S1 (the head, including the receptor binding domain, or RBD, in addition to an N-terminal domain) and S2 (the stem, which contains multiple sub-domains, including a fusion peptide, a central helix, two heptad repeats, a transmembrane domain, and a cytosolic tail). S1 is in charge of binding to the host cell receptors while S2 is in charge of fusing the viral and host membranes. The high glycosylation level of the S protein is shown to play a role in folding as well as evading the host’s immune system [44]. The S protein can be in a closed or open state (Figure 3C). The open state is necessary for binding to the receptor.

The RBD domain is responsible for binding to the ACE2 and starting infection (Figure 3D,E). The core of this domain mostly consists of B sheets which are stabilized through several disulfide bonds. The core is highly conserved. The external subdomain is mostly dominated by loops which are stabilized through a disulfide bond. The interaction between the RBD domain and ACE2 is highly dominated by hydrophilic interactions and hydrogen bonding across the interface (Figure 3D,E).

**Figure 4:**
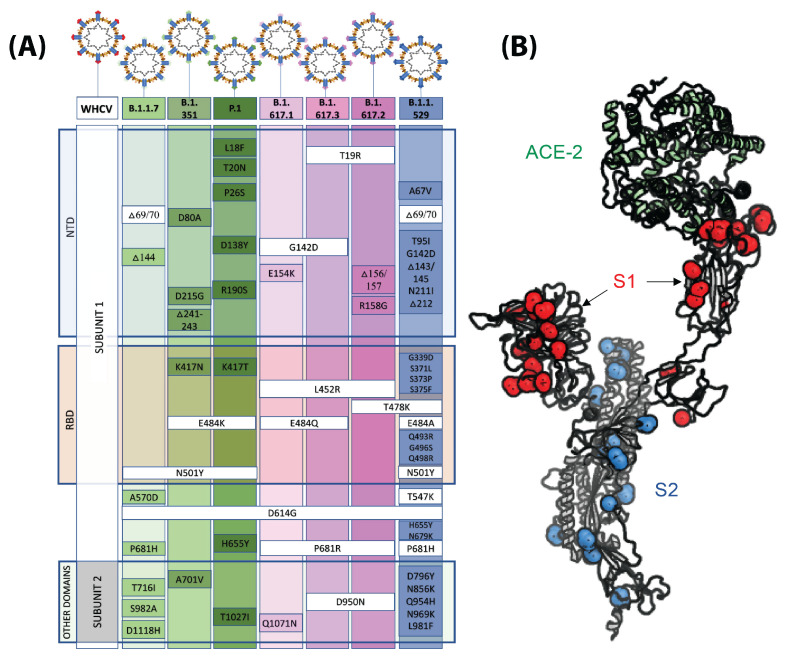
(**A**) observed mutations in the spike protein among variants of concern in SARS-CoV-2. (**B**) Structural location of these variants. The spheres indicate the locations of observed mutations in either S1 or S2 subdomain [46].

One hallmark of RNA viruses including SARS-CoV-2 is their rapid mutation rates [46]. Many mutations are silent, in that they do not change amino-acid residues and, thus, result in no functional consequences. However, there are many examples of mutations that can affect the structure and function of viral proteins and confer evolutionary advantages in terms of increased pathogenesis, faster transmission, or higher survival rates. Over the 2 years since the beginning of the COVID-19 pandemic, many new variants have developed, some of which have higher transmission rates or increased pathogenicity. In many of these cases, we can observe mutations in the S protein that can explain these observations. Figure 4A shows the location of these mutations and their type. Figure 4B highlights the location in the structure of the S protein.

## 4. Electrode Design

### 4.1. Electrode Materials

The three-electrode setup is the most commonly used setup for EIS measurements (as it is for other voltametric and amperometric measurements). This setup is composed of a working electrode (WE), a counter (auxiliary) electrode (CE), and a reference electrode (RE). Surface modifications and impedance analysis is carried out on the WE. Novel WE modification methods are the main component of interest in many electrochemical studies. The RE is a low-impedance electrode designed to establish a stable reference potential in the electrochemical system. Without a stable reference potential, we cannot be confident that changes in the impedance signal are due to WE surface changes and not due to drift. Most REs are developed using a combination of Ag and AgCl materials and function as quasi-reference electrodes given the presence of chloride in all biofluids. The CE is a low-resistance electrode (typically Pt or Au) which allows current flow between the CE and WE and enables impedance measurements.

**Figure 5:**
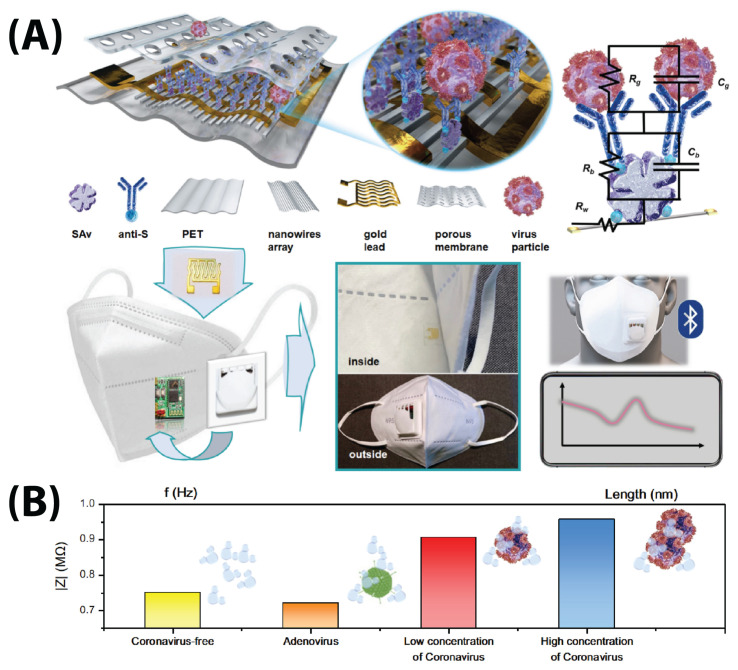
(**A**) Xue et al. developed a mask-integrated sensor using interdigitated Au nanowires [69]. (**B**) The impedance response increases with the presence of viral particles. This is reprinted with permission from Elsevier [69].

The large majority of reviewed papers utilized a three-electrode system [9,48,49,50,51,52,53,54,55,56,57,58,59,60,61,62,63,64,65]. However, in EIS, because impedance is the main parameter, it is possible to operate with a two-electrode system [66,67,68,69]. In theory, two-electrode systems with a WE and a combination RE/CE are inherently less stable with repeated measurements than their three-electrode counterparts [17]. A dedicated CE is important for providing a low-resistance path for current to flow to (1) prevent signal attenuation due to the high-resistance RE and (2) protect the RE from high currents, which can damage it and its ability to establish a stable reference potential [17]. However, the two-electrode systems reviewed demonstrated limits of detection (LOD) comparable to the best of their three-electrode counterparts, reaching past the femtomolar and femtogram/milliliter ranges [66,67,68,69]. This impressive performance is likely due to extreme measurement ranges (Soares et al.), which can artificially improve LOD and the use of highly specific, small aptamers for binding (Ramanathan et al.) (the impact of these two factors will be discussed later) [66,67]. Of the four two-electrode papers, only Xue et al. measured repeatability within the same electrode, demonstrating an acceptable 4.7% (*n* = 3) relative standard deviation (RSD) (Figure 5) [69].

**Figure 6:**
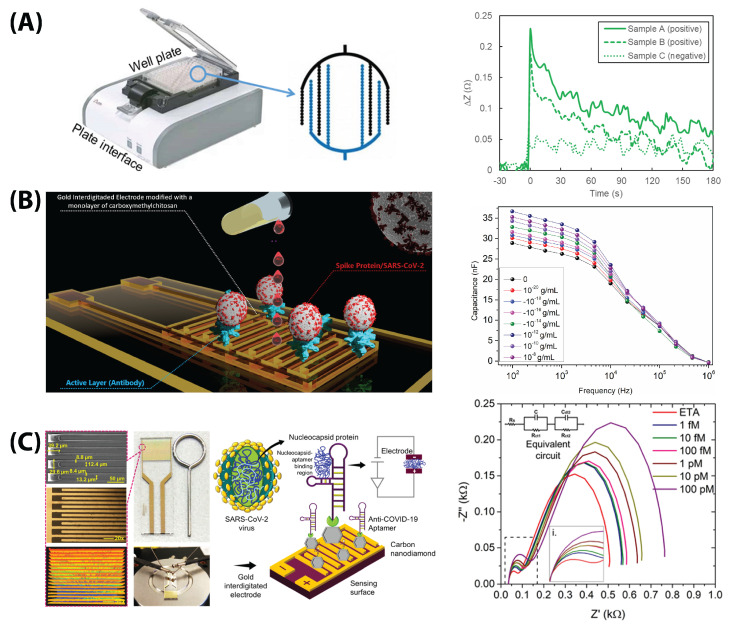
(**A**) Early on in the SARS-CoV-2 pandemic, Rashed et al.’s interdigitated electrode system was based on specialized plates and plate readers, and performed single-impedance readings [68]. (**B**) Soares et al. developed an antibody-based Au-Au interdigitated electrode modified with carboxylmethyl chitosan to detect the presence of S protein [66]. (**C**) Ramanathan et al. purchased interdigitated electrode and modified them with diamon nanopowder and an aptamer to detect the N protein [67]. Reprinted with permission from Elsevier [66,67,68].

Carbon-based electrodes are desirable because of their low cost and versatility in functionalization. Abrego-Martinez et al., Wu et al., Lorenzen et al., and Brazaca et al. used electrodeposition to add AuNPs to the surface of their carbon electrodes, while Hussein et al. added WO_3_ by electroplating [9,48,54,57,64]. Soares et al. (Figure 6B) added carboxymethyl chitosan to their carbon WE and Li et al. and Torres et al. added glutaraldehyde to their carbon electrodes [56,66,70]. Other groups opted to use the popular electroactive polymer poly(3,4-ethylenedioxythiophene) polystyrene sulfonate (PEDOT:PSS) on top of existing carbon and Au electrode material [57,69,71].

### 4.2. Electrode Form Factors

#### 4.2.1. Commercial Screen-Printed Electrodes

Electrodes have taken a variety of different form factors. They are typically two-dimensional, flat electrodes made of different materials. This form factor was popularized by commercially available screen-printed electrodes such as the Dropsens electrodes manufactured by Metrohm. Some groups opted to take advantage of this existing manufacturing technology and built their sensors by modifying the surface of existing commercially available sensors [48,53,54,55,61,62,64,65,67,68].

Tepeli et al. (Dropsens), Soto and Orozco (Dropsens), Wu et al. (Zensor), Hussein et al. (Gamry), Abrego-Martinez et al. (Biodevice Technology), and Sharif et al. (Dropsens) purchased fully integrated electrodes [48,53,54,61,62,64]. Soares et al., Ramanathan et al. (Figure 6C), Rashed et al. and Lasserre et al. utilized specially commissioned single or interdigitated electrodes [55,66,67,68].

#### 4.2.2. Self-Designed Electrodes

Others opted to manufacture electrodes themselves, and low-cost materials such as paper and thin films stood out from other materials due to their flexibility and versatile chemical properties (although Ashur et al. used conventioanl silicon lithography) [9,49,50,52,56,58,59,60,69,70,71]. Researchers developed and patterned their electrodes onto these low-cost substrates with various methods. Pola et al. and Ali et al. used aerosol jet printing and Salahandish et al. used a flatbed microprinter to deposit electrode material [49,60,71]. Xue et al. used nanoscale soft printing to deposit Au wire, while Li et al. grew hydrothermal ZnO wire onto paper [56,69]. Nicoliche et al. used oven pyrolization to generate single-carbon WEs [58]. Perdomo et al. and Brazaca et al. used screen printing to deposit conductive ink onto their substrates [9,59]. Wax printing was also used to define microfluidic channels in paper-based devices [52,63]. Lorenzen et al. used a steel mesh as a sensing-platform substrate [57].

While all these substrates are relatively low-cost, two different paradigms of fabrication methodology emerge: one driven by cost-efficiency and scalability (i.e., wax and screen printing), and one focused on precision manufacturing (i.e., photolithography and hydrothermal growth); they tend to be inversely related. The advantages and disadvantages of each are outlined in Table 1. However, both paradigms seem to enable well-controlled batch electrode fabrication, which allows electrode miniaturization, lower required sample volumes, and reduced cost compared to qRT-PCR.

One must also consider spatial resolution when selecting a fabrication method. Aerosol jet-printing techniques achieve feature sizes of 10–100 μm [72]. The resolution of screen-printed electrodes depends on the contact angle of the specific ink and substrate, as well as the pseudoplasticity of the ink [73]. In lithography, the wavelength of the exposure system and the type of photoresistance are the two determining factors for resolution [74]. By varying wavelength, feature sizes from less than 1 nm to higher than 100 nm can be achieved: ion beam lithography results in feature sizes less than 1 nm, electron beam lithography systems can achieve feature sizes of less than 10 nm, and optical lithography can be used for feature sizes higher than 100 nm [74].

New form factors have surfaced more recently due, in large part, to the adaptability of EIS to different substrates. Rashed et al. (Figure 6A) modified a well-plate electrode with an RBD protein to detect anti-SARS-CoV-2 antibodies, and Ali et al. built a PDMS-molded microfluidic flow channel on top of 3D microprinted reduced graphene oxide structures [49,68]. Perhaps most interestingly, Xue et al. developed an impedimetric face-mask-integrated sensor for detecting the S protein in exhaled breath aerosols (Figure 5) [69].

### 4.3. Immobilization Protocols

Perhaps the most critical step in fabricating an EIS sensor is functionalizing it with the appropriate capture element. Before doing so, a cleaning step is sometimes used to remove impurities from the surface of the electrode, which may hinder functionalization.

Au has traditionally been employed as an electrode material due to its inertness and excellent thermodynamic stability [75]. Various Au cleaning procedures, such as chemical and electrochemical cleaning, have been reported in the prior literature [76]. To eliminate environmental pollutants from the Au surface, an oxidizing substance such as sulfuric acid can be used to chemically clean the surface [77]. Electrochemical cleaning can also be performed, where the electrodes are placed in acid solution and voltammetric cycling is performed [78]. These cleaned electrodes are then ready for immobilization.

**Figure 7:**
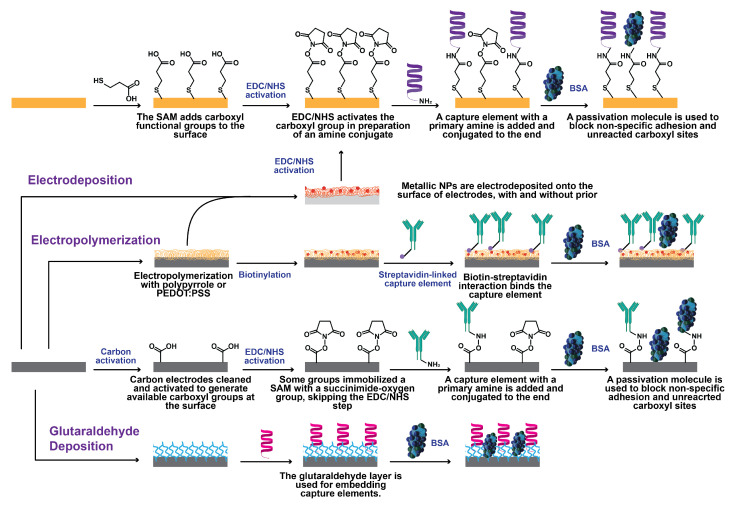
The fabrication schemes of different electrodes. Nearly all electrodes begin with a Au or carbon base, but have different modification steps. Au is most often used in conjunction with a thiol group to take advantage of thiol–Au chemistry, either with a thiol-based crosslinker (as shown here) or directly with a thiol-based capture element (such as an aptamer). Electropolymerization is used to add metal nanoparticles onto the surface of the electrodes. EDC/NHS steps can be applied either directly to a monolayer formed on Au or onto functional groups present in carbon electrodes. Glutaraldehyde is used for embedding capture elements in a matrix nonspecifically. BSA is used to block the electrode surface and prevent nonspecific interaction.

One of the most popular tools for immobilization is thiol-Au chemistry (Figure 7). This typically forms a monolayer onto the surface of the electrode which can uniformly orient the capture elements. Ashur et al., Soto and Orozco, Lasserre et al., and Abrego-Martinez et al. deposited thiolated antibodies, peptides, and aptamers directly onto Au surfaces [48,50,55,61]. The thiol-Au chemistry was also used in conjunction with the popular N-ethyl-N’-(3-(dimethylamino)propyl)carbodiimide/N-hydroxysuccinimide (EDC/NHS) chemistry used for activating carboxylic acids to form amide bonds with primary amines. In these WEs, researchers first add a thiolated acid to an Au electrode, where the thiolated end interacts with Au to form an orderly monolayer which exposes an aligned carboxylic acid for EDC/NHS chemistry to take place [9,49,62,64,67].

The EDC/NHS chemistry has also been used apart from acid monolayers. Perdomo et al. electrodeposited para-aminobenzoic acid on a carbon electrode to utilize EDC/NHS chemistry [59]. Soares et al., Pola et al., and Zaccariotto et al. used EDC/NHS chemistry on a carbon electrode with already-exposed carboxyl groups to immobilize capture elements [60,65,66].

There are many other methods for capture-element immobilization. Wu et al. and Xue et al. used the tried-and-tested biotin-streptavidin chemistry to functionalize their electrodes (Figure 7) [64,69]. In addition, outside of specific linking chemistries, several groups also used nonspecific adhesion by simply drop casting the capture elements onto predominantly carbon electrodes (Rashed’s group drop casted on an Au electrode nonspecifically, and Ramanathan et al. drop casted onto diamond nanopowder) [57,58,67,69,71]. Ehsan et al. immobilize a monolayer of 1-pyrenebutanoic acid succinimidyl ester via their Van der Waals forces, which results in the same exposed O-pyrene group as the end result of EDC/NHS coupling [52]. Avelino et al., Torres et al., and Li et al. immobilized their capture elements in glutaraldehyde matrixes [51,56,70]. Hussein et al. and Sharif et al. captured their analytes with a molecularly-imprinted polymer, thus not requiring specific linking chemistries [53,54].

After surface modification, the electrodes are typically blocked with a passivating molecule to prevent nonspecific adhesion or interaction with activated carboxyl groups. It was most common to use a low percent (0.005–1%) of bovine serum albumin (BSA), since it is large and neutrally charged at physiological pH [51,52,55,57,58,59,64,65,70,71]. Alternative molecules that were used include ethanolamine, mercaptohexanol, 3% milk, and proprietary blocking solution [48,56,60,61,67,68].

### 4.4. Sample Volume

The design and size of the electrodes is closely related to the sample size that can be added to the electrode. Often, fully integrated sensors (where the WE, RE, and CE are all integrated onto one surface) will require between 1–10 μL of sample, with only three groups using samples sizes of 50 μL [9,48,49,50,51,53,54,58,61,67,68,70,71]. Perdomo et al. were able to use only 0.3 μL of sample to operate their sensor (however, they used a more standard 50 μL of measurement buffer containing a redox marker) [59]. The interdigitated electrodes made by Soares et al., Ramanathan et al., and Rashed et al. required 250, 20, and 50 μL samples, respectively [66,67,68].

Further reduction in sample volumes makes testing easier on patients and makes frequent and widespread testing more feasible. In addition, this does seem possible; groups did not optimize sample volume, but simply reported the sample volume used. At some point, there are diminishing returns in reducing sample volume, however, so a minimal-but-reasonable amount of sample should be used (i.e., 10 μL is approximately a pinprick of blood—further reduction in sample volumes may not have practical advantage).

## 5. Electrical Detectors and Detection Parameters

**Figure 8:**
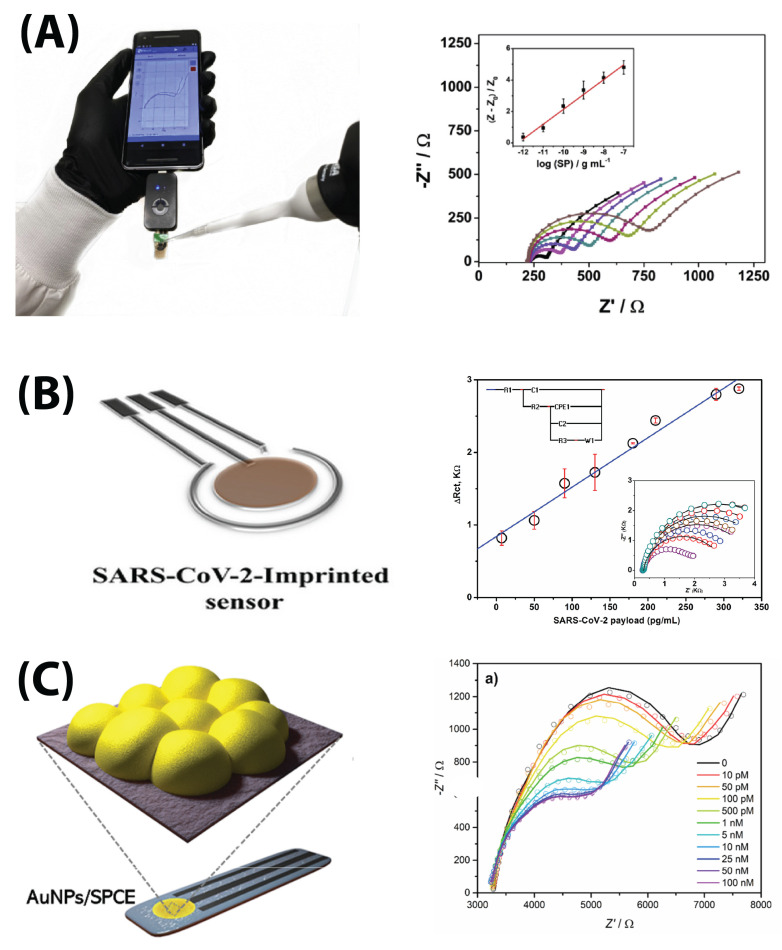
A selection of fully integrated EIS sensors for COVID-19. (**A**) A paper-based carbon–carbon–Ag/AgCl ink electrode by Torres et al. [70]. (**B**) A carbon–carbon–carbon commercial Gamry electrode purchased and modified with carbon nanotubes and tungsten trioxide by Hussein et al. [54]. (**C**) A carbon–carbon–Ag/AgCl commerical electrode modified with AuNP by Abrego-Martinez et al. [48]. These are reprinted (adapted) with permission from Elsevier and American Chemical Society [48,54,70].

In order for these electrodes to provide actionable information, they need to be used in conjunction with a device that (1) generates an AC signal to perturb the sample and (2) reads the resulting impedance values. These signal readers can also contain electronics for data output to smart devices and/or data processing (Figure 8A). Some groups used existing devices such as the PalmSens4 and Metrohm Autolab PGSTAT204, while others used traditional benchtop electrochemical workstations [51,52,53,56,58,64,65]. Additionally, yet other groups developed their own electrical detectors, often with a focus on portability and use at the point of care [49,51,55,59,60,61,69,70,71]. Most electrodes tended to fall within the range of a few centimeters, while devices tended to be slightly larger (a few centimeters more) [49,50,51,54,55,59,60,61,62,69,70,71].

To perturb the system, each of the devices reviewed measured a range of frequencies to generate a semicircular Nyquist plot which could be fitted to a Randles equivalent circuit (Figure 1). The range of frequencies varied, but they typically began scanning around 10–100 kHz and completed their scan in the 0.01–0.001 Hz range [9,48,49,50,51,52,53,54,55,56,57,58,59,60,61,62,63,64,65,66,67,70,71]. These measurements were typically performed in a solution containing ferricyanide and ferrocyanide as redox markers. Most groups applied a current with amplitudes between 5 mV and 10 mV (although some applied amplitudes in the range of hundreds of millivolts) and applied a DC bias of 0.1–0.2 V.

It is worth noting that more/fewer measurement points can be taken within the same range of frequencies. Salahandish et al. took both 7-point and 50-point measurements from 1–1400 Hz and found that the 7-point measurement was sufficient to generate a Nyquist plot to create an accurate calibration curve [71]. Fewer measurement points can simplify analysis and make measurements significantly faster. Some groups, such as Xue et al. and Rashed et al., use single-point impedance to generate signals from their sensors [68,69]. The disadvantage of measuring fewer frequency points is the potential to miss important information which could be revealed with a more detailed frequency sweep. Additionally, higher resolution frequency sweeps provide more data, which can be conducive to feeding ML algorithms to further increase diagnostic speed and accuracy.

## 6. Analyte Detection

There are two main classes of COVID-19 EIS sensor: (1) viral tests and (2) antibody tests. The choice of antigen is significantly impacted by which type of sensor is desired. Of the 24 reviewed papers, 19 were viral tests and only five sought to detect antibodies. Tests were created to detect three different portions of the SARS-CoV-2 viral particle (Figure 2B): (i) the S protein (sometimes specifically the S1 subunit), (ii) the RBD of the S1 subunit, and (iii) the N protein (Figure 3B). A helpful summary of all reviewed papers is found in Table 2.

The sensors are exposed to varying concentrations of analytes, as well as interfering or non-specific molecules. This affects the surface of the electrode, changing the equivalent circuit and the resulting Nyquist plot (Figure 1).

### 6.1. S Protein Detection

The S protein was detected by six different groups with a range of LODs from the picogram to the femtogram-per-milliliter ranges [9,59,60,66,69]. Popular sources of anti-S antibody were Sinobiological and Abcam.

**Figure 9:**
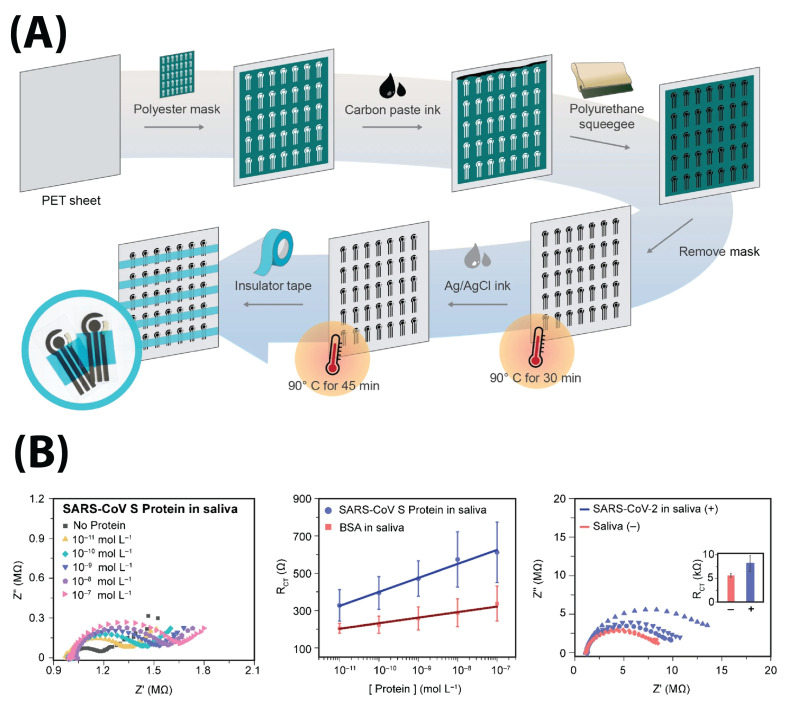
(**A**) The screen-printing fabrication process of Brazaca et al. for their carbon–carbon–Ag/AgCl electrodes [9] (**B**) The sensor response demonstrates increasing impedance parameters with increasing analyte concentration. Reprinted with permission from Springer Nature under the Creative Commons BY-SA 4.0 license.

Perdomo et al., Brazaca et al., and Soares et al. used anti-S protein antibodies bound to the electrode via EDC/NHS coupling [9,59,66]. Perdomo et al. and Brazaca et al. used three-carbon electrode systems to detect the S protein in PBS redox buffer [9,59]. Perdomo et al. achieved a limit of detection (LOD) of 1.065 fg/mL after finding linearity at concentrations between 1–20 fg/mL [59]. Brazaca et al. detected an LOD of 83.7 pg/mL when calibrated between 10 pM and 0.1 μM (Figure 9) [9].

Soares et al. used a two-Au-electrode sensor to detect S protein samples in diluted viral solution without redox marker at up to and LOD of 0.179 fg/mL (testing a range of 10 zg/mL–10 ng/mL) [66]. It is notable they use capacitance as a detection parameter instead of the more common $R_{ct}$ (Figure 6B) [66].

Xue et al. used an anti-S protein antibody immobilized via biotin-streptavidin onto PEDOT:PSS nanowires to detect the S protein in a mask-integrated sensor (Figure 5). They obtained an LOD of 7 pfu/mL (0.35 pfu/L of air) in an aerosolized solution (mimicking breathed-out saliva) [69]. Pola et al.’s attempt at S1 protein detection resulted in an aerosol jet-printed carbon electrode functionalized with polyclonal anti-S protein antibodies which had a 110.38 ± 9.00 pg/mL LOD when exposed to a range of 1–1000 ng/mL S1 protein [60]. r Although Brazaca et al. and Pola et al. report an LOD four orders of magnitude higher than Perdomo et al. and Soares et al., this does not necessarily indicate an inferior process. This is because LODs are determined by a calculation based on either (i) the perceived intercept between the linear response and nonlinear response or (ii) the standard deviation and slope of a regression line. Both methods have objectivity issues. A bi-linear response (i.e., Ramanthan et al., Figure 6C) could be misinterpreted as nonlinearity, therefore preventing full characterization of the electrode response. Variability in a manufacturing technique could, similarly, have a negative effect on LOD calculations; Soares et al.’s 10-fold lower LOD compared to Perdomo et al.’s LOD is likely due to the use of precision photolithography for fabrication and treatment, over the latter’s use of basic screen-printing technology [59,66].

Torres et al. (Figure 8A) and Tepeli et al. used biologically inspired design to detect the S protein using ACE2 and CD147 transmembrane glycoproteins as capture elements, since they are two receptors in the body that bind the SARS-CoV-2 virus [62,70]. Torres et al. were able to use ACE2 receptors immobilized with glutaraldehyde to detect S protein at a concentration of 1.39 pg/mL after testing a range of 100 fg/mL–100 ng/mL in human saliva [70]. Tepeli et al. immobilized both ACE2 and CD147 onto sensors and found LODs of 299.30 and 38.99 ng/mL (respectively) after testing in ranges of 700–7000 and 500–5000 ng/mL [62]. It is notable that Torres et al.’s carbon screen-printed system demonstrated a 100-fold superior LOD compared to Torres et al.’s commercial Au electrode system, although this, again, may be due to the latter’s testing range being significantly higher. Lasserre et al. used anti-S1 protein aptamers to detect the S1 protein, but did not demonstrate data, only correlating the results with a negative/positive result [55].

These biologically inspired sensors demonstrate performances comparable to the more traditional antibody-based sensors. The major advantage here is that existing biological structures such as ACE2 and CD147 are typically more familiar (protein structure, manufacturing, chemical stock, etc.) early on in an endemic compared to antibodies against the novel infectious agent (i.e., anti-S protein antibodies took time to produce at scale for research). Therefore, biologically inspired molecules can enable a more rapid response to a new disease than waiting for reliable antibodies to be produced. However, specific-antibody-based sensors are still important, since biologically inspired capture elements are likely to include nonspecific interactions native to healthy individuals.

### 6.2. RBD Detection

Five groups detected the RBD protein and achieved LODs at the pico- and femtogram levels [48,52,60,61,65]. Of the five groups, two groups used peptide sequences and DNA aptamers as capture probes [48,61]. The three remaining groups used anti-S1 antibodies to capture the RBD protein, since the RBD is the primary binding site of the S protein to cell receptors such as ACE2 [52,60,61,65]. A variety of different suppliers sourced the different capture elements, including Genscript.

**Figure 10:**
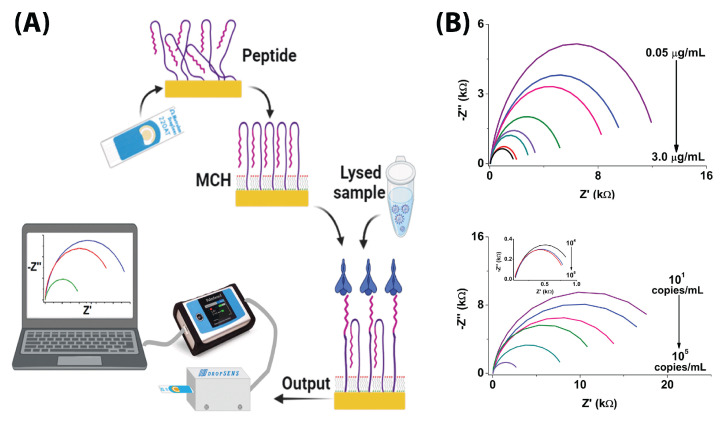
(**A**) The fabrication process of Soto and Orozco. They used an Au–Au–AgCl commercial screen-printed electrode from Dropsens and modified them with capture peptides [61]. (**B**) The sensor demonstrated a negative response, where increasing concentrations of S protein and lysed viral particles decreases the impedance signal. Reprinted with permission from Elsevier [61].

Soto and Orozco used a thiol-immobilized 23-amino acid peptide sequence which mimics the ACE2 receptor to capture the RBD protein with an LOD of 0.01 copies/mL in redox buffer with potassium nitrite (Figure 10) [61]. They tested the modified commercial Au electrode in a linear range of 100–1000 copies/mL [61]. Abrego-Martinez et al. also used a thiolated aptamer to detect up to 1.30 pM (66 pg/mL) RBD with a linear range of 10 pM–25 nM in PBS redox solution (Figure 8C) [48].

Zaccariotto et al. detected RBD with an anti-S1 antibody immobilized by EDC/NHS onto a glassy carbon disk electrode [65]. They tested in the range of 0.16–40 μg/mL with an LOD of 150 ng/mL in a PBS redox buffer [65]. Ehsan et al. and Pola et al. printed (by hand and by CNC) graphene electrodes and modified them with anti-S1 antibodies which demonstrated 0.25 fg/mL and 22.91 ± 4.72 pg/mL LODs for RBD when tested in ranges of 0.25 fg/mL–1 ng/mL and 1–1000 ng/mL [52,60]. Pola et al. tested in PBS redox buffer, while Ehsan et al. tested in redox solution (without PBS) [52,60].

The performance of the RBD detectors is comparable to the performance of the S protein detectors. There was no significant difference in terms of selectivity between the different sensors (many groups did not report a selectivity difference, and if they did, the parameters used varied).

### 6.3. N-Protein Detection

The N protein is not involved with the SARS-CoV-2 virus entering cells, but it is critical for packaging the RNA genome inside the viral capsid [79]. The N protein is enclosed in the viral capsid and is not accessible from outside an intact viral particle. However, the N protein is abundantly expressed during infections, and is easily detected in infected cells. Four different EIS sensors measured N-protein levels spiked into artificial solution [51,64,67,71]. Wu et al. went further, validating their tests in artificial saliva, and Avelino et al. validated their test against qRT-PCR with real nasophrayngeal/oropharyngeal swab samples [51,64]. Similar to the RBD and S protein sensors, these sensors reached LODs in the range of femtograms.

Salahandish et al. and Wu et al. used anti-N-protein antibodies from Genscript and Vazyme to detect N proteins in PBS redox buffer [64,71]. Salahandish et al. detected up to 116 fg/mL when calibrating from 1–10,000 pg/mL with their carbon + graphene@PEDOT:PSS electrodes [71]. Wu et al. achieved an LOD of 6 pg/mL and a range of 0.1–100 ng/mL with an AuNP-modified commercial carbon electrode from Zensor [64].

Instead of antibodies, Ramanathan et al. used an aptamer immobilized onto an Au interdigitated electrode via a silanization reaction to obtain an LOD of 0.389 fM (approximately 0.443 pg/mL) with a linear range of 1 fM–100 pM (Figure 6C) [67]. Avelino et al. took a different route, using an amino-modified primer to detect the nucleocapsid gene instead of the protein, achieving an LOD of 258.01 copies/μL with a linear range of 800–4000 copies/μL [51].

As with the RBD sensors, the N-protein aptamer sensors show comparable performance to the traditional antibody-based N-protein sensors, and the difference in LOD is difficult to compare, since the tested ranges and variability are so different.

### 6.4. Whole-Virus Detection

Three groups opted to detect entire viral particles instead of only proteins expressed on the surface of those particles. Ashur et al. used Traut’s reagent to thiolate an anti-S-protein antibody for immobilization on a three-electrode polytetrafluoroethylene-based Au electrode [50]. They did not detect the SARS-CoV-2 virus, but developed a pseudovirus which expressed the S protein on its surface [50]. They were able to detect a range of 104 to 109 viral particles/mL, and when detecting S protein in solution, achieved an LOD of 15 ng/mL (500 pM) [50].

Hussein et al. (Figure 8B) did not use a traditional capture element for their detector; they instead casted a layer of 3-aminophenol monomer mixed with a human sample of the SARS-CoV-2 virus, then washed out the virus to leave a viral imprint [54]. They were able to achieve an LOD of 57 pg/mL; while no range was reported, they tested the sensor at up to 320 pg/mL in redox buffer [54]. El Sharif et al. did the same with a N-hydroxymethyl acrylamide monomer with an LOD of 0.69 pfu/mL when tested in 0.477–0.845 pfu/mL; they were one of the few groups who tested in real samples with saliva biofluid [53].

This molecular imprinting technique has been used to develop NPs selective for the COVID-19 virus [80]. Thomaz et al. investigated the performance of recombinant antibodies compared to molecularly imprinted NPs for impedimetric detection of the SARS-CoV-2 RBD protein and found that they can have a binding performance comparable to high-affinity anti-RBD recombinant antibodies [80]. Such molecularly imprinted NPs have the advantage in stability and production capacity, two issues which have not yet been resolved with conventional or recombinant antibodies.

Sensors developed with a whole-virus detection mindset show comparable LODs to protein-detecting EIS sensors [50,53,54]. Additionally, testing for the detection of whole viral particles more closely mimics the physiological condition, which is beneficial for the practical translation of the technology.

### 6.5. Antibody Tests

The presence of antibodies is an indicator of immunity against an infection, likely caused by a previous or current infection. Antibody test currently requires blood draws, and therefore is a target for translation into low cost, less invasive EIS sensors. Five groups attempted to detect antibodies using the corresponding antigen, obtained from a variety of sources, including laboratory-synthesized antigens [49,56,57,58,68].

Ali et al. detected anti-S-protein antibodies and anti-RBD antibodies by immobilizing S proteins and RBD onto 3D-microprinted Au structures coated with reduced graphene oxide via EDC/NHS chemistry [49]. They obtained an LOD of 2.8 fM and 16.9 fM for anti-S protein and anti-RBD antibodies in ranges of 1 fM–30 nM and 1 fM–20 nM in PBS buffer [49]. Li et al. immobilized RBD onto zinc oxide nanowires grown onto carbon ink for an LOD of 0.4 pg/mL when tested between 1 ng/mL–1 μg/mL in PBS redox buffer [56].

Nicoliche et al., similarly, used the S protein dropcasted onto a pyrolyzed graphitic paper electrode to detect antibodies against the S protein in PBS redox buffer [58]. Rashed et al. used single-point impedance for their sensor; see Figure 6A [68]. They used RBD nonspecifically attached to Au interdigitated electrodes embedded into the bottom of a well plate to detect a range of anti-S-protein antibodies in 3% milk buffer [68]. However, neither group reported an LOD or a detection range. Lorenzen et al. immobilized truncated N proteins onto AuNP-modified PEDOT:PSS, but tested saliva dilutions instead of precise concentration values [57].

The field seems to prefer rapid viral tests over rapid antibody tests. For one, there are so few tests looking for antibodies, and three of the tests do not report significant quantified data. However, as previously mentioned, there is a largely unexplored field of rapid, low-cost EIS devices for antibody tests that would prove extremely helpful for determining immunity and exposure to viruses such as SARS-CoV-2.

## 7. Practical Considerations

If the aim of EIS device for SARS-CoV-2 monitoring is convenient, low-cost, rapid detection of COVID-19 infections, then there are other critical considerations beyond simply the analytical performance of the sensor.

### 7.1. Shelf-Life

The shelf-life of a sensor is important for logistical reasons. Many biological sensors are vulnerable to degradation when not stabilized in solution or exposed to heat. This means that manufacturing location and transportation are critical considerations.

Sensor stability was tested in seven of the papers reviewed (only 29%), but the rigor of the test was not standardized; some groups tested for a single day, while others tested for several months [51,54]. Generally, electrodes tended to remain viable after around two to three weeks when kept at a cold temperature (4 ${}^{{}^{\circ}}$C) before use [48,61,67,70]. Xue et al. and Hussein et al. kept their sensors at room temperature and found stability remaining after 3 days and 2 months, respectively [54,69].

Electrode type and manufacturing process seems to have an impact on stability as well. For example, Xue et al.’s 3-day viability was for Au nanowire sensors with PEDOT:PSS polymerized onto it, with S protein adhered [69]. Hussein et al.’s 2-month viability was for a robust, electroplated electrode with a polymeric vial imprint as a capture element [54]. The latter sensor is more robust due to material alone—the metal portion has greater mechanical strength, and the capture element does not denature like the devices produced by Xue et al. [54,69]. Non-biological capture elements allow sensors to last far longer than other sensors; while these sensors can last two months without special care, multiple biological capture-element sensors last only 2–3 weeks with special care and refrigeration [48,51,54,61,70]. The investigation of sensors built with such non-biological capture elements, therefore, warrants increased attention due to their resiliency.

### 7.2. Reproducibility

We will define the manufacturing reproducibility of an electrode system as the interelectrode variability and the test-to-test reproducibility. Consistency in manufacturing is important if the end goal is widespread COVID-19 testing; batch to batch, each electrode should produce similar results within a particular margin of error. This is the goal of “calibration-free” electrodes, that are electrodes so consistently manufactured that individual calibration of each electrode is not required for accurate readout.

The intraelectrode variability of an electrode system should also be low. This is not as important for single-use systems (where the interelectrode variability is much more important), but for any system that is intended to be used multiple times, it is imperative that the electrodes provide consistent results.

As with shelf-life, both inter- and intraelectrode variability were not always quantified. However, the majority of papers that did quantify and report this data did so in the form of the RSD in measured concentration.

Only nine groups measured interelectrode RSD. Brazaca et al., Hussein et al., Soto and Orozco, and Wu et al. manufactured and measured the RSD of three identical electrodes per process, finding RSDs of, 5.12%, 3%, 2.2%, and 0.4–3.1% RSD, respectively [9,54,61,64]. Ali et al. measured six different electrodes for their S protein and RBD electrodes, finding 3.7 and 2.7% RSD [49]. Torres et al. and Ehsan et al. tested 10 different electrodes with a 6.8% RSD and <10% RSD, and Avelino et al. did not report the number of electrodes they tested, but reported 1.31% RSD [51,52,63]. Pola et al. reported the worst results for their RBD and S1 protein LODs at 20.60% and 8.15% RSD (*n* = 3) [60].

Intraelectrode RSD was measured by nine groups. With the same counts as interelectrode reproducibility, Ali et al. measured 3.2 and 0.25% RSD for their electrodes and Brazaca et al. measured 4.51% RSD, while Hussein et al. just reported “high repeatability” [9,49,54]. Avelino et al., and Tepeli et al. did not report the number of measurements performed, but reported <1% RSD, and 4.5% (ACE2) and 4.03% (CD147) RSD [51,62]. Soto and Orozco reported a 4.1% RSD with five measurements (Figure 10), while Torres et al. measured 5.3% RSD with 21 measurements on the same electrode [61,70]. Xue et al. did not quantify variance, but just noted that after repeated measurements, 96.3% of the signal was retained [69]. Again, Pola et al. reported the poorest results, showing a 15% signal drive after 5 measurements [60].

Salahandish et al. found a coefficient of variance of 6.9%, which is a different method of measuring variability [71].

All the measurements taken demonstrate inter- and intraelectrode RSDs of around 5% or less. This is excellent for sensor development and within a reasonable margin of error. It is also likely and feasible that better manufacturing tolerances would further decrease the margin of error. Intraelectrode reproducibility is mainly dependent on operator skill and standardization of immobilization and post-processing steps. More sophisticated techniques that require precision machinery (such as photolithography) tend to be more reproducible than more basic, hand-powered techniques such as screen printing. Material choice also matters—for example, the uniformity of an Au electrode is dependent on the sputtering technique, while a graphene layers’ uniformity changes based on graphene production and deposition. Material processing also matters—the deposition of AuNPs depends on electrode surface area and electrical parameters, and the cleaning and activation of graphite/graphene is highly dependent on the chemical and electrical procedures used. As shown by these numerous studies, these can be mostly consistent if performed by the same group of people on the same materials—however, at a larger scale, small variations in technique can have significant effects on device performance and characteristics.

### 7.3. Measurement Buffer

All electrochemical measurements, from voltammetry to amperometry and EIS, require charge carriers in measurement solution. Bodily fluids will typically contain sufficient amounts of these charge carriers in the form of salt ions and trace metal ions—for example, the heme core of hemoglobin molecules can function as an redox probe. However, when characterizing and evaluating the performance of an electrochemical sensor in vitro, large concentrations of a reversible redox molecule are typically used. This is to ensure that impedance signals are due to changes at the electrode surface, and not because there is a lack of charge carriers in the solution.

All but three of the devices were tested in a ferricyanide/ferrocyanide buffer. These two molecules are readily reduced and oxidized into each other, but are not naturally present in biological fluid. The difference between this redox buffer and biological fluid is not negligible. Complete testing of electrochemical sensors should include testing in real or simulated biological fluids without the use or addition of extra reagents, including unnatural redox probes such as ferricyanide/ferrocyanide. Some sensors are designed to be only incubated in biological fluid and then measured in a reversible redox buffer [50,58]. While this will result in better and more interpretable signals (especially if used in concert with washing steps to remove non-specific and unbound molecules), it requires additional work and specialized chemicals for each test, which increases testing complexity and limits the technology’s reach into under-resourced communities and its promise as a rapid and widespread testing screening methodology.

### 7.4. Antifouling

One of the biggest hurdles for EIS sensors is surface fouling through nonspecific adsorption or other surface-altering damage. Nonspecific adsorption of proteins, peptides, and other organic matter onto the surface of the electrode will cause changes in the response of the electrode, leading to false positive results. In addition, if the selective surface of the electrode is damaged, the performance of the electrode can significantly worsen.

BSA was used as an antifouling agent in many reported studies. Due to its widespread availability, abundance, and simplicity, BSA is often used as a monolayer to passivate surfaces—to coat the devices with a known molecule and, thus, prevent nonspecific adsorption on the electrode surface [81]. As previously mentioned, another group used 3% milk to achieve the same effect, while others used small molecules such as mercaptohexanol and ethanolamine to quench exposed reactive sites [48,61,67,68]. Smaller molecules can cover the electrode surface more thoroughly (large proteins such as BSA may leave gaps which cannot be fully covered due to steric hindrances), but can be more expensive, difficult to use, or pose potential cross-reactivity issues.

## 8. Summary and Future Perspectives

### 8.1. Summary

Electrochemical impedance spectroscopy is a powerful tool in the fight against highly contagious diseases such as COVID-19 due to its relatively simple setup, low cost of deployment, and rapid result output. Many groups have attempted to develop EIS electrodes for detecting the SARS-CoV-2 or portions of it, and have been successful. However, the practical application and scalability of these electrodes is still unknown. Devices that require microfabrication are still relatively expensive to produce and deploy, but offer high reliability and performance, while devices made from simpler means can be made at scale, though with lower reliability. To date, Torres et al. has created the most complete EIS device for COVID-19 detection, and it is in the clinical-trial testing stage [70]. This paper-based device is relatively robust, but can still be produced at scale. If commercially successful, it would cause a paradigm shift in the way the COVID-19 pandemic is fought worldwide, and bring a swifter end to this battle.

### 8.2. Standardization

If the field of EIS sensor technology is to become competitive with the well-established gold standards of the day, a standardization of testing and methodology is imperative. The selection of a standard analysis parameter should be chosen well, and nonstandard parameters should be convertible and related to the standard parameter (it does seem that $R_{ct}$ is the most common parameter). Detection limit and range should be a standard requirement for analysis. As much as possible, testing should be performed on actual viral particles, or else researchers should attempt to convert LODs and linear ranges for viral subparticles (i.e., S, S1, RBD, and N proteins) into values for viral loads (i.e., ng/mL of SARS-CoV-2). Nonspecific interference should be analyzed quantitatively (perhaps with a binding or selectivity constant). In addition, interelectrode and intraeletrode variances should be reported along with clear stability reports. Without this standardization, EIS sensors will continue to be evaluated in many different ways, and comparing will be difficult.

### 8.3. Novel Form Factors

Form factor is another area of growth for EIS-based sensors. Currently, manufacturing processes still largely produce planar electrodes. While paper is a flexible material, current paper-based devices typically do not take full advantage of the unique flexibility of paper. Yarns and textiles are yet another materials horizon, and more and more researchers are looking into the use of carbon nanotubes as an interesting and unique material.

### 8.4. Novel Data-Analysis Tools

Machine learning is the science of learning a new task or identifying a pattern from a given set of input data [82]. Machine-learning algorithms have been widely used in COVID-19 related applications for data analysis and disease diagnosis. However, the adaptation of ML in impedimetric sensors using impedance spectroscopy data has been extremely limited [83]. The typical calibration process of impedimetric sensors involves selecting an equivalent circuit model for the EIS spectrum and reducing the problem to a linear regression between an electrode parameter (such as Δ$R_{ct}$) and analyte concentration. Machine-learning approaches can be used to automate the equivalent circuit selection process, thus removing human errors. Additionally, ML tools can capture multi-parameter relationships between the high-dimensional EIS data and the analyte concentration, reducing the possible loss of information in one variable calibration [30,84].

Impedance spectroscopy data along with ML have been used in battery characterization applications [85]. Zhang et al. collected 20,000 EIS spectra of commercial lithium-ion batteries and used a Gaussian process regression model to predict the health and the effective remaining lifetime of the battery [85]. Classification models have been used to classify the equivalent circuit model for each EIS spectrum and to estimate elements’ values [84,86,87].

**Figure 11:**
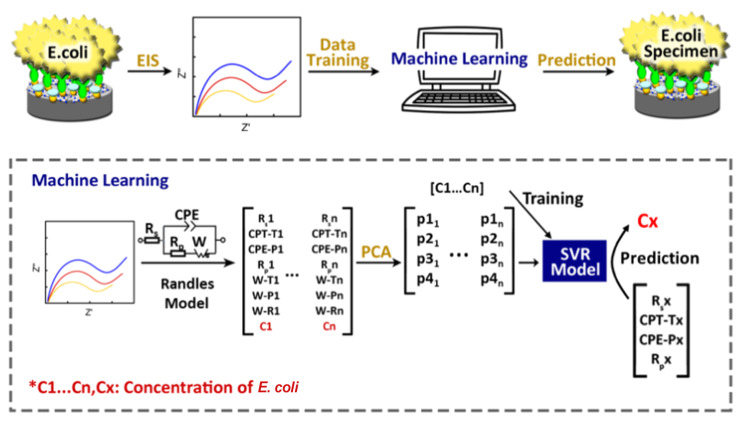
A graphic representation of how ML can work for EIS. Taken from [30] © The Electrochemical Society. Reproduced by permission of IOP Publishing Ltd. All rights reserved.

ML algorithms are utilized to improve the performance of impedimetric sensors [88]. In 2020, Xu et al. developed an impedimetric sensor for the detection of *Escherichia coli* using ML (Figure 11) [30]. They first extracted the equivalent circuit elements’ values using numerical methods. After reducing the dimensionality through principal component analysis (PCA), a support vector regression model was used to determine the concentration of *E. coli*. Unlike the traditional method, in which a linear calibration curve is generated from a single parameter at different analyte concentrations, they used all the parameters of the equivalent circuit as inputs for their model [30].

Additionally, ML algorithms are applied to different fields of medicine, including disease diagnosis, prediction, and classification, to achieve high accuracy, sensitivity, and specificity [83]. Technologies based on ML had an impact on COVID-19 screening and treatment, contact tracing, modeling disease spread, and developing drugs and vaccines [89,90,91]. At the interface of ML and COVID-19 impedimetric sensors, ML is able to increase the sensitivity and accuracy of sensors by reducing reliance on fallible human observation and capturing hidden and/or complex interactions between the input and output [30,84]. However, one of the main common challenges in all these applications is the acquirement of a large and reliable dataset for training ML models.

### 8.5. Novel Capture Probes and Variant Detection

Finally, the potential of alternative capture probes is beginning to be realized. To date, antibodies have been the dominant capture probe for biosensor design. Despite excellent selectivity, they suffer from issues such as short half-life, high batch-to-batch variation, and stringent storage and transport requirements, which limit clinical translation.

Synthetic and designed peptides, proteins, and nanobodies can be very selective towards our target analytes, while remaining more stable over time compared to traditional antibodies. Stability when dry and without refrigeration is very important for a detection device intended for use in low-resource and remote regions, where refrigerator-free bulk storage and collection is crucial. Additionally, synthetic peptides and nanobodies can be produced more cheaply and quickly than the traditional method of incubating and purifying antibodies from living cells or animals—another important consideration for the industrialization and scaling of these devices.

One area of research is the development of capture probes which can specifically detect select variants of concerns. These probes can be instrumental for epidemiologic studies of the spread of the disease and guiding future policy decisions [92]. As shown in Figure 4, the core of the S protein is conserved and most observed variants in the S protein are observed on the surface. These surface residues are what directly interact with the capture probes; therefore, the generation of variant-specific capture probes seems reasonable. These probes can be generated using experimental tools, state-of-the-art computational design techniques, and advanced artificial-intelligence-driven algorithms.

### 8.6. Final Comments

The field of EIS-based sensors for healthcare has great promise. For rapid COVID-19 diagnosis, sensors developed by Torres et al. showed the greatest promise, while Perdomo et al. and Ehsan et al. developed similar sensors with competitive LODs [52,59,63,70]. Now, researchers must focus on thoroughly characterizing their sensors so that comparative analyses can lead to improvements. Thorough sensor characterization should include standard LOD calculations, concentration reporting, repeatability testing, and interference testing. Real biological samples should also be tested, as much as possible. As sensors become more powerful, collaboration with experts in other fields will pave the way for widespread adoption of these life-saving technologies in healthcare.

## Figures and Tables

**Table 1:** A summary of different fabrication methods.

Fabrication Method	Advantages	Disadvantages
Aerosol jet printing	Simple to perform and iterate designs. Modification of standard office inkjet printer. Highly detailed features.	Can require multiple passes to deposit sufficient material. Modifying printers can be challenging.
Electrodeposition	Existing protocols for surface modification and deposition. Good adhesion for surface nanoparticles.	Restricted to electroactive nanoparticles. Requires expensive metal chloride reagents. Slower prototyping.
Electropolymerization	Existing protocols for surface modification and polymerization. Wide variety of polymers. Controllable and tunable.	Slower prototyping.
Flatbed microprinter	High resolution. Rapidly iterable.	Requires suitable ink. Expensive and specialized equipment.
Hydrothermal wire growth	Fine, conductive, high surface area.	Highly sensitive to environmental changes in temperature and humidity. Resource intensive.
Pyrolization	Low material cost. Simple technique.	Cannot control pattern. Requires high-temperature ovens. Only produces carbon materials.
Photolithography	High resolution. Allows printing metal. Well-established protocols. Established production pipeline.	Requires high-skilled labor. Expensive and time consuming. Microfabrication facilities unavailable to low-resource setting.
Screen Printing	Simple to perform. Cost efficient. Inexpensive.	Surface area can be difficult to control. Screens need to be replaced and cleaned regularly. Limited spatial resolution.

**Table 2:** Comparison of technical performance of the reviewed EIS-based sensors for diagnosis of COVID-19.

Electrode Type	Working Electrode	Sample Volume	Response Time	Analyte	Capture Element	Linear Range	Detection Limit	Real Sample	Reference
Fully integrated	Carbon + AuNP	5 μL	–	RBD protein	Aptamer	1 × 10${}^{-11}$–25 × 10${}^{-9}$ M (5.08 × 10${}^{-10}$–1.27 × 10${}^{-12}$ g/mL)	1.30 × 10${}^{-12}$ M (66 pg/mL)	–	[48]
Fully Integrated	Au + rGO flakes	30 μL	15 s	S1 protein Ab RBD protein Ab	S1 protein RBD protein	S1: 1 × 10${}^{-15}$– 30 × 10${}^{-9}$ M RBD: 1 × 10${}^{-15}$–20 × 10${}^{-9}$ M	S1: 2.8 × 10${}^{-15}$ M RBD: 16.9 × 10${}^{-15}$ M	–	[49]
Fully Integrated	Au	15 μL	45 min	SARS-CoV-2 pseudo types VSV particle	S1 protein Ab	10${}^{4}$–10${}^{9}$ VSV/mL	15 ng/mL (500 pM)	–	[50]
Fully Integrated	Carbon Ink + AuNP	10 μL	30 min	S protein Ab	S1 protein	10 × 10${}^{-11}$ to 10 × 10${}^{-7}$	3.16 pM (83.7 pg/mL)	–	[9]
Fully Integrated	Carbon Ink	0.3 μL	5 min	S protein	S protein mAb	1–20 fg/mL	1.065 fg/mL	–	[59]
Fully Integrated	Carbon + Graphene + PEDOT:PSS	50 μL	30 min	N protein	N protein Ab	1–10,000 pg/mL	116 fg/mL	Nasopharyngeal swab	[71]
Fully Integrated	Carbon Ink	4 μL	2 min	S protein	ACE2	10 fg/mL–100 ng/mL	2.18 fg/mL	Saliva, Oropharyngeal and Nasopharyngeal swabs	[70]
Fully Integrated (Dropsens)	Au	50 μL	15 min	RBD protein S protein Lysed COVID-19 particles	Targeting peptide	10^2^–10^3^ copies/mL	0.01 copies/mL	Nasopharyngeal swabs	[61]
Fully Integrated (Dropsens)	Au	10 μL	45 min	S protein	ACE2 receptor CD147 receptor	ACE2: 700–7000 ng/mL CD147: 500 ng–5000 ng/mL	ACE2: 299.30 ng/mL CD147: 38.99 ng/mL	Oropharyngeal and Nasopharyngeal swabs	[62]
Fully Integrated (Dropsens)	Au	50 μL	2 min	SARS-CoV-2	Molecularly-imprinted polymer	3.0–7.0 log10 PFU/mL	4.9 log10 pfu/mL	Saliva	[53]
Fully Integrated (Gamry)	Carbon + CNT + Tungsten Trioxide	5 μL in 2.95 mL PBS	3 min	Whole Virus - Human-obtained sample, grown in cell line	Virus-imprinted monomer (3-aminophenol)	Up to 320 pg/mL tested	57 pg/mL	Nasopharyngeal swabs	[54]
Fully integrated (Paper, wax printed)	Carbon Graphene ink	10 μL	5 min	RBD Protein	S1 IgG Ab	0.25 fg/mL–1 ng/mL	0.25 fg/mL	Nasopharyngeal swabs	[52]
Fully Integrated (Paper)	Carbon + Zinc-Oxide Nanowires	3 μL	15 min	S1 protein Ab	N protein	Tested: 10 × 10${}^{-9}$–1 × 10${}^{-6}$ g/mL	0.4 pg/mL	Serum	[56]
Interdigitated	Au + Carboxylmethylchitosan	Immersion	10 min	S protein Real samples	anti-S protein Ab	Protein: × 10${}^{-20}$– × 10${}^{-8}$ g/mL Real Sample: 7 × 10${}^{-3}$–7 × 10${}^{5}$ PFU/mL	0.179 fg/mL	Isolated real virus	[66]
Interdigitated	PEDOT:PSS	Aerosol	5 min	S protein	Antibody	–	7 pfu/mL, 350 pfu/mL (air)	Aerosolized porcine transmissible gastroenteritis virus	[69]
Interdigitated	Au + diamond nanopowder	20 μL	5 min	N protein	N protein aptamer	1 × 10${}^{-15}$–1 × 10${}^{-10}$ M	0.389 fM	–	[67]
Single Electrode	Tin-doped indium oxide + polypyrrole + AuNP	2 μL	15 min	SARS-CoV-2 Nucleocapsid Gene	Amino-modified primer	800–4000 copies/μL	258.01 copies/μL	Nasopharyngeal	[51]
Working Only	Carbon (Pyrolyzed Graphitic Paper)	6 μL	30 min	COVID-19 Ab	S protein	–	–	–	[58]
Working Only	Carbon + AuNP	10 μL	40 min	N protein	N protein murine Ab	0.1–100 ng/mL	6 pg/mL	Saliva	[64]
Working Only	Glassy Carbon + rGO	10 μL	–	RBD protein	S1 protein Ab	0.16–40 μg/mL	150 ng/mL	Saliva	[65]
Working Only	Graphene powder in ethyl cellulose (custom ink)	15 μL	30 min	RBD protein S1 protein	S protein (Rabbit PAb)	1–1000 ng/mL	22.91 ± 4.72 pg/mL (RBD) 110.38 ± 9.00 pg/mL (S1)	–	[60]
Working Only	Thin-Film Au Electrode	–	15 min	S1 protein	Aptamer	Not tested	Positive/negative only (80 ng/mL), *n* = 8	Nasopharyngeal and Oropharyngeal swabs	[55]
Working Only	Steel Mesh + PEDOT-AuNP	Immersion	30 min	SARS-CoV-2 Ab	Truncated	20 × 10${}^{-3}$–2.5 × 10${}^{-3}$ dilutions	–	Serum	[57]
Well-plate	Au	50 μL	None	SARS-CoV-2 antibody	RBD protein	0.1, 1.0, 10 mg/mL standards tested	–	–	[68]

## Data Availability

Not applicable.
